# Remdesivir Use During Pregnancy: Findings From the Prospective COVID‐19 International Drug Pregnancy Registry and Narrative Literature Review

**DOI:** 10.1155/jp/8700344

**Published:** 2026-07-06

**Authors:** Diego F. Wyszynski, Cheryl Renz, Aprille Espinueva, Lee P. Shulman, EunYoung Lee, Andrew Fitzgibbon, Gina Brown

**Affiliations:** ^1^ Thermo Fisher Scientific, London, UK, thermofisher.com; ^2^ Thermo Fisher Scientific, Waltham, Massachusetts, USA, thermofisher.com; ^3^ Gilead Sciences Inc., Foster City, California, USA; ^4^ Division of Clinical Genetics, Feinberg School of Medicine, Northwestern University, Chicago, Illinois, USA, nwu.edu.cn

**Keywords:** antiviral therapy, drug safety, maternal health, perinatal outcomes, pregnancy complications

## Abstract

**Background and Aims:**

Remdesivir (RDV), an inhibitor of coronavirus replication including SARS‐CoV‐2, has been approved for the treatment of COVID‐19. Nevertheless, its use during pregnancy lacks specific efficacy and safety data due to the exclusion of pregnant patients from clinical trials. Authorization for compassionate use in pregnant women was granted during the conduct of the Phase 3 clinical trials in adult patients hospitalized with moderate or severe COVID‐19. Available evidence from prior studies suggests that no consistent safety concern has been identified, although available data remain limited. The most commonly reported adverse event (AE) is transaminitis, which rarely necessitates treatment discontinuation.

**Methods:**

Safety data for RDV exposure during pregnancy were collected by the COVID‐19 International Drug Pregnancy Registry (COVID‐PR) between December 2021 and November 2023. Information was obtained through self‐administered online questionnaires completed during pregnancy and up to 1 year after live birth, with medical record corroboration when available.

**Results:**

Nine women reported a total of 15 AEs, with an additional seven AEs reported in three infants. Ten of these AEs were considered serious and included a life‐threatening hypersensitivity episode. No consistent pattern of AEs suggestive of a specific safety concern was identified.

**Conclusions:**

This study contributes additional observational safety data regarding RDV use during pregnancy. However, due to the small size of the study population and various study limitations, definitive conclusions about the safety of this treatment in pregnant populations cannot be drawn.

**Plain Language Summary:**

RDV is a drug used to treat COVID‐19 by blocking the virus from multiplying in the body. However, since pregnant women were not included in early clinical trials, we did not have much information about how safe and effective this drug is for them. During the pandemic, some pregnant women with severe COVID‐19 were allowed to receive RDV through compassionate use programs, and our study is aimed at collecting data on their experiences. We looked at 23 pregnant women who took RDV between December 2021 and November 2023. Out of these, eight were followed through the end of their pregnancy, resulting in seven live births and one miscarriage. Some women and their babies experienced side effects, with the most common being temporary increases in liver enzyme levels, and one woman had a serious allergic reaction. Overall, no consistent pattern of adverse pregnancy or infant outcomes was identified. However, because only a small number of pregnancies were available for analysis and many participants were lost to follow‐up, larger studies are needed to better understand the safety of RDV use during pregnancy.

**Key Points**

• Evidence regarding remdesivir (RDV) use during pregnancy remains limited, particularly for first‐trimester exposure.
• Among reported events with available follow‐up, most were classified as nonserious.
• Transaminitis was the most frequently reported adverse event (AE), whereas one serious hypersensitivity reaction was observed.
• Among pregnancies with follow‐up data available, no consistent pattern of adverse maternal, fetal, or neonatal outcomes was identified.
• Given the small sample size and substantial loss to follow‐up, definitive conclusions regarding RDV safety during pregnancy cannot be drawn.

## 1. Introduction

RDV is an inhibitor of SARS‐CoV‐2 RNA polymerase acting as a nucleotide analogue [1–3]. Upon administration, it rapidly converts into GS‐441524, a nucleoside analogue, and GS‐443902, a pharmacologically active nucleoside triphosphate [1, 4, 5]. The latter mimics adenosine triphosphate (ATP) and competes with endogenous ATP for incorporation into SARS‐CoV‐2 RNA, leading to delayed chain termination and inhibition of viral replication [1, 5].

Originally developed for Ebola virus, which belongs to the Filoviridae family, RDV has demonstrated effectiveness against multiple RNA viruses, including Pneumoviridae, Paramixoviridae, and other Filoviridae [1, 6]. Notably, RDV inhibits SARS‐CoV‐1, MERS‐CoV, and bat coronaviruses [1], and its potency against SARS‐CoV‐2 infection spurred interest in its use for COVID‐19 treatment [1, 7].

Although RDV undergoes rapid metabolism, its metabolites exhibit an extended plasma half‐life of approximately 35 h. Consequently, intravenous RDV administration is required once daily, achieving steady state within 5 days [1]. Data on RDV pharmacokinetics during pregnancy remain limited. The International Maternal, Pediatric, Adolescent AIDS Clinical Trials Network 2032 study, involving 12 pregnant participants, reveals comparable levels of RDV metabolite GS‐704277 in pregnant and nonpregnant populations, with reduced RDV and GS‐441524 levels during pregnancy. A physiologically based pharmacokinetics model aligns with observed data, though intracellular peripheral blood mononuclear cell concentrations may differ during pregnancy, and could have dosing implications [8].

Simulation studies suggest that pregnancy‐related increases in glomerular filtration and renal tubular secretion may enhance elimination of RDV′s major extracellular metabolite, GS‐441524. Changes in plasma protein concentrations could further alter unbound RDV and metabolite levels, potentially requiring dose adjustments to achieve exposures comparable to those in nonpregnant populations [6]. However, a recent clinical study reported no meaningful differences in plasma concentrations of RDV or its major metabolites between pregnant and nonpregnant patients, indicating that pregnancy‐specific dose adjustment may not be necessary [9].

Excluding pregnant patients from clinical trials has led to scant drug efficacy and safety data in this population [1, 10]. Available RDV evidence is limited to the second and third trimesters, with no available first‐trimester information [10]. Available data are derived primarily from postmarketing registries, cohort studies, case series, and case reports, many of which lack comparison groups [6, 10]. Incomplete obstetric and neonatal data further complicate data interpretation [2]. Robust evidence on RDV′s safety and efficacy in pregnancy is urgently needed [10]. In the absence of randomized controlled trials, well‐designed real‐world evidence studies offer an alternative to address challenges in enrolling adequate numbers across trimesters.

Current guidelines recommend treating pregnant patients with COVID‐19 similarly to nonpregnant counterparts [11, 12], cautioning against withholding treatment solely due to pregnancy or lactation [11]. As anatomical and physiological changes during pregnancy heighten vulnerability to respiratory infections, treating such infections becomes crucial, though a cautious stance on RDV in pregnancy persists [1, 13]. These considerations underscore the importance of continued evaluation of the safety and effectiveness of RDV use during pregnancy [1, 13].

In this paper, we present pertinent RDV treatment data from the initial 12 months of the COVID‐19 International Drug Pregnancy Registry (COVID‐PR), alongside an overview of available efficacy and safety data concerning RDV use during pregnancy.

## 2. Methods

The COVID‐PR (NCT05013632, EUPAS42517) is a global postmarketing cohort study designed to gather safety data from pregnant and recently pregnant women treated with monoclonal antibodies or antiviral drugs to manage mild, moderate, or severe COVID‐19 during pregnancy [14, 15]. The study received ethics approval from the Western Institutional Review Board–Copernicus Group Institutional Review Board (WCG IRB) (20214420). Women were recruited into the study as soon as possible after treatment. All participants provided written informed consent, including consent for publication of deidentified data. Potential participants were made aware of the study through drug labeling, the internet and social media, and relevant scientific meetings and publications. Efforts were made to recruit participants from underrepresented populations through targeted social media outreach and geographically diverse recruitment strategies. Participants completed online questionnaires throughout pregnancy and up to 1 year postpartum. Participant‐reported data encompass both maternal and neonatal outcomes, supplemented by relevant medical records when available. Causality assessments are conducted by COVID‐PR personnel, with independent dysmorphologists engaged for serious adverse fetal outcomes. The study is being conducted in countries where the study drugs are accessible and is expected to continue for 5 years. The primary objective is to recruit a minimum of 200 pregnancies for each investigated COVID‐19 therapy. Recruitment for RDV began on December 1, 2021, and data included in this report were collected through November 30, 2023.

The COVID‐PR was originally planned to include comparator groups of women not treated with the study‐specific drugs [14]. However, due to low participant numbers, this was not feasible, and analyses were restricted to descriptive statistics. All analyses were prespecified and included maternal, neonatal, and infant outcomes commonly associated with pregnancy. In addition, AE data were collected. For baseline characteristics and AEs, the number and percentage of women responding to each category are presented.

Within the stipulated timeframe, a total of 23 participants who underwent RDV treatment during pregnancy, all hailing from the United States, enrolled in the COVID‐PR registry. An overview of participant baseline characteristics is presented in Table 1. Several baseline variables contained substantial missing data, which should be considered when interpreting the findings. Participant ages ranged from 19 to 42 years, with a mean age of 30 years (standard deviation: 6.76). Among participants with available demographic information, most identified as White, reported at least a high school education, denied current tobacco or alcohol use, reported daily vitamin supplementation, and described themselves as being in good prepregnancy health. Two participants disclosed existing comorbidities (arrhythmia and asthma), potentially influencing COVID‐19 severity. Roughly three‐quarters of the participants underwent RDV treatment during their second or third trimester of pregnancy.

**Table 1:** Baseline characteristics of COVID‐PR participants treated with remdesivir.

Characteristic	Number	%
Total number of participants	23	100.0
Age (years)		
18–20	2	8.7
21–25	6	26.1
26–30	5	21.7
31–35	5	21.7
36–40	3	13.0
41–45	2	8.7
Ethnicity		
White/Caucasian	10	43.5
Black/African American	1	4.3
Hispanic, Latina, or Spanish origin	3	13.0
Missing data	9	39.1
Highest level of education attained		
Graduate/professional	1	4.3
College	9	39.1
High school	4	17.4
Missing data	9	39.1
Employment status at onset of pregnancy		
Employed, on maternity leave	1	4.3
Employed, full‐time (35+ hours/week)	7	30.4
Not employed, not looking for work	4	17.4
Not employed, looking for work	2	8.7
Missing data	9	39.1
Tobacco use		
None	8	34.8
Yes, before this pregnancy	3	13.0
Yes, before and during this pregnancy	1	4.3
Missing data	11	47.8
Alcohol use during pregnancy		
Never	9	39.1
Once per month	3	13.0
Missing data	11	47.8
Gestational timing of treatment		
First trimester	5	21.7
Second trimester	9	39.1
Third trimester	9	39.1
Vitamin use during pregnancy		
Daily	11	47.8
4–6 times/week	1	4.3
Missing data	11	47.8
Prepregnancy body mass index		
18.5–25.9	3	13.0
26.0–30.0	2	8.7
> 30.1	7	30.4
Missing data	11	47.8
Self‐reported prepregnancy health		
Excellent	5	21.7
Very good	5	21.7
Good	4	17.4
Missing data	9	39.1
Existing medical conditions at onset of pregnancy		
None	7	30.4
At least one condition	4	17.4
Arrhythmia	1	4.3
Asthma	1	4.3
Cancer	1	4.3
High blood pressure	1	4.3
Hypothyroidism	1	4.3
Other	1	4.3
Missing data	12	52.2
Healthcare coverage at onset of pregnancy		
Yes	11	47.8
No	3	13.0
Missing data	9	39.1

## 3. Results

As of November 30, 2023, seven participants had given birth, one experienced a spontaneous abortion at an unspecified gestational age, and fifteen were lost to follow‐up. Among these seven live births, two were delivered via cesarean section.

Of the seven infants, their birthweights spanned from 2778 to 3912 g, with a mean birthweight of 3175 g and a standard deviation of 406 g. Four of these infants were delivered at a gestational age of 39–42 weeks, two at 37–38 weeks, and the seventh baby was born prematurely at 36 weeks and 5 days. Additional details regarding this case are provided below.

### 3.1. Safety Outcomes

Details of the AEs reported are provided in Table 2.

**Table 2:** Adverse events reported for remdesivir treatment during pregnancy. COVID‐PR study from December 2021 to November 2023.

Adverse events	Number (%)		Serious adverse event	Number of days between treatment and start of adverse event	Possibly related to remdesivir treatment^1^
**Maternal**					
Number of participants who reported at least one AE^2^	9		—		—
Total number of adverse events	15		—		—
Dyspnea	2	(13.3)			
Case 1^a^			Yes	0	Yes
Case 2^b^			No	108	Yes
COVID‐19 reinfection	1	(6.7)	No	Not calculated^3^	No
Facial swelling^a^	1	(6.7)	Yes	0	Yes
Hyperhidrosis	1	(6.7)	No	0	Yes
Hypersensitivity^a^	1	(6.7)	Yes	0	Yes
Hypertension	1	(6.7)	No	Not calculated^3^	Not reported
Hypotension^c^	1	(6.7)	No	0	Yes
Hypoxia^a^	1	(6.7)	Yes	0	Yes
Irregular heart rate^b^	1	(6.7)	No	108	Yes
Oligohydramnios	1	(6.7)	Yes	56	Not assessable
Pharyngeal swelling^a^	1	(6.7)	Yes	0	Yes
Preeclampsia	1	(6.7)	Yes	150	No
Premature delivery	1	(6.7)	No	79	No
Spontaneous abortion^c^	1	(6.7)	Yes	15	Yes
**Infant**					
Number of infants with at least one reported AE^2^	3		—		—
Total number of adverse events	7		—		—
Ear infection	2	(28.6)			
Case 1			No	470	No
Case 2^d^			No	458	No
Congenital long QT syndrome^e^	1	(14.3)	Yes	151	No
COVID‐19 infection^d^	1	(14.3)	No	292	No
Decreased fetal heart rate^e^	1	(14.3)	Yes	Not calculated^3^	No
Prematurity^d^	1	(14.3)	Yes	79	No
Umbilical cord around neck^e^	1	(14.3)	No	Not calculated^3^	No

^1^According to the COVID‐PR safety physician. ^2^Some participants and infants reported more than one adverse event, superscript letters indicate how these were grouped in the individual participants lettered a, b, c, d, or e. ^3^Could not be calculated as the start date of the adverse event was not reported.

### 3.2. Maternal Outcomes

In total, nine participants reported a total of 15 AEs during the treatment period. Dyspnea was the only preferred term that emerged more than once. This is not particularly surprising, given that it is a common symptom of both pregnancy and COVID‐19. Seven of the 15 AEs, occurring in three study participants, were classified as serious.

Regarding six of these seven serious AEs (87.5%), the lack of comprehensive information precluded definitive establishment of a clear connection to RDV treatment, although a relationship to RDV treatment could not be excluded. For the seventh serious AE, an assessment could not be rendered due to insufficient details provided.

Among the remaining seven AEs classified as nonserious, four were identified as possibly associated with RDV treatment.

### 3.3. Neonatal Outcomes

Seven AEs were reported involving live‐born infants. Although three of these AEs were classified as serious, none of them were determined to be possibly related to RDV treatment.

Additional insights into the serious AEs are elaborated upon in the subsequent section.

### 3.4. Case 1

A 26‐year‐old pregnant woman, carrying twins, experienced a spontaneous abortion 16 days after receiving a single administration of RDV. The gestational age at the time of RDV exposure was not reported. On January 10, 2022, she developed symptoms of COVID‐19, including cough, difficulty breathing, fever, loss of smell, nausea or vomiting, persistent chest pain or pressure, runny nose, sore throat, and fatigue. The highest recorded temperature was 100°F. On January 11, 2022, the diagnosis of COVID‐19 was confirmed and, 2 days later, she was treated with a single dose of RDV. On the day of RDV administration, the patient also experienced an episode of hypotension, which resolved after a period of 7 days. On January 29, 2022, she had a spontaneous abortion of both conceptuses. Despite multiple attempts, important information such as a past medical history of pregnancy losses, use of concurrent medications, presence of pregnancy‐related comorbidities, results of chromosomal analysis, and any uterine abnormalities were not provided.

#### 3.4.1. Commentary

Although the timeline potentially hints at a causal role for RDV, the 16‐day interval between exposure and the event weakens this possibility. Notably, COVID‐19 itself has been linked to spontaneous abortion, making it a significant confounding factor [16, 17]. The increased risk of spontaneous loss or preterm delivery in twin pregnancies also adds complexity [18]. Fetal mortality rates in twin pregnancies are approximately threefold higher than those in singleton pregnancies and are strongly associated with preterm birth [19]. Concurrent hypotension′s potential influence on placental blood flow is noteworthy. Although direct causality between the medication used to treat COVID‐19 and pregnancy loss is unlikely, a temporal association between RDV exposure and the event cannot be excluded. Hence, this AE was classified by the COVID‐PR safety physician as possibly related to RDV treatment. However, it is crucial to acknowledge that multiple factors, including COVID‐19 infection and the nature of twin pregnancies, might have contributed to the outcome.

### 3.5. Case 2

A 20‐year‐old female participant experienced a life‐threatening hypersensitivity episode with symptoms of dyspnea, facial and pharyngeal swelling, and hypoxia on the same day as RDV treatment, leading to immediate discontinuation. The reported symptoms strongly suggest an allergic reaction to the medication.

#### 3.5.1. Commentary

Considering the event timeline and RDV′s established safety profile, classifying these events as possibly related to RDV treatment is reasonable. However, it is important to note that the potential impact of COVID‐19 infection on exacerbating or contributing to the allergic reaction symptoms cannot be dismissed. Comprehensive clinical information, encompassing the patient′s full medical history, concomitant medications, relevant diagnostic parameters, and treatment administered, is required for a thorough causality assessment.

### 3.6. Case 3

A 30‐year‐old female participant developed oligohydramnios 56 days after RDV treatment. No additional information was provided regarding possible risk factors contributing to oligohydramnios, such as maternal hypertension, preeclampsia, diabetes, dehydration, hypoxia, or placental abnormalities.

#### 3.6.1. Commentary

Due to the lack of relevant information and the multifactorial nature of oligohydramnios, it is challenging to establish a relationship between this AE and RDV treatment. It is unlikely that RDV played a causal role. Other factors, such as underlying maternal conditions or gestational complications, may be more significant contributors. A comprehensive evaluation of the participant′s medical history and additional clinical data are essential to better comprehend the potential causes of oligohydramnios in this instance.

### 3.7. Case 4

Five months after maternal RDV exposure, a female neonate, born to a 22‐year‐old participant, displayed reduced fetal heart rate during delivery and was diagnosed with congenital long QT syndrome at birth. Notably, the mother had a history of arrhythmia, introducing a confounding factor.

#### 3.7.1. Commentary

Given the information presented, it is unlikely that the observed decreased fetal heart rate during labor resulted from the mother′s COVID‐19 treatment during pregnancy. Instead, it is more plausible that the neonate′s diagnosis of congenital long QT syndrome was responsible. Congenital long QT syndrome is typically hereditary or idiopathic and has no direct link to RDV exposure. The 5‐month gap between RDV exposure and the AE further weakens the likelihood of a direct association. Based on the available data, the congenital long QT syndrome was considered unrelated to RDV treatment. A comprehensive evaluation of the neonate′s medical history, genetic factors, and clinical information is necessary to better understand the factors influencing the neonate′s condition.

### 3.8. Case 5

A female neonate, born to a 39‐year‐old participant, was delivered prematurely via cesarean section at 36 weeks and 5 days gestation. The mother had received RDV treatment 50 days prior to delivery. The primary reason for the cesarean section was the mother′s history of previous cesarean delivery. Although previous COVID‐19 infection and polypharmacy could have played a role, this is unlikely.

#### 3.8.1. Commentary

After careful consideration, there appears to be no plausible mechanism connecting the reported AE to RDV treatment, and a clear temporal association is absent. Thus, this AE was determined to be unrelated to RDV treatment. Other factors, including the participant′s medical history, the need for cesarean section, and the known associations between COVID‐19 infection and both preterm birth [20] and cesarean section [21], are more likely to have contributed to these outcomes.

## 4. Discussion

### 4.1. Principal Findings

The COVID‐PR is an ongoing study aiming to enroll a minimum of 200 pregnancies for each medication under scrutiny. As of November 2023, a total of 22 AEs were reported in the COVID‐PR study. Among these, 15 AEs were reported by 9 study participants, whereas an additional 7 were reported in 3 of the 7 delivered infants. Among the mothers, seven AEs were categorized as serious, with six cases in which a causal link to RDV could not be definitively established or ruled out. In the infants, three AEs were serious, but they did not seem to be associated with RDV treatment, and plausible alternative causes were identified. No consistent pattern of AEs was identified. Although these findings are generally consistent with observations reported in other studies of RDV use during pregnancy, the available data remain too limited to support definitive conclusions regarding safety [2, 9, 22–27].

### 4.2. Study Limitations

Study limitations must be acknowledged, as they may affect the validity and interpretation of the COVID‐PR findings.

1. These results are based on a limited number of participants and data regarding COVID‐19 disease severity, which may influence pregnancy outcomes, were not available.
2. The majority of participants in the study were White and thus may not accurately reflect the population that acquired COVID‐19 in the United States, who were disproportionately Black or Hispanic.
3. A notable proportion of participants were lost to follow‐up, though the study protocol includes provisions for ongoing attempts to reengage these individuals, although the impact of this missing outcome data on study validity remains uncertain.
4. Information on COVID‐19 vaccination status was not collected for the participants. As vaccination may significantly influence disease severity, maternal and pregnancy outcomes, and potentially the occurrence of AEs, the absence of this information represents a major confounding factor.
5. The study relied upon self‐reported questionnaire data, which was only partially confirmed by medical records. Therefore, the data are at substantial risk of reporting bias, recall bias, and outcome misclassification. The lack of standardized clinical validation further weakens the reliability of the data.
6. The study did not collect information regarding the timing of infection in relation to the trimester of pregnancy. Instead, the timing of RDV administration acted as a proxy. Given that the course of COVID‐19 infection varies widely, this means that in some cases there could have been a considerable delay between infection and RDV treatment. This could impact the likelihood and type of AEs subsequently reported, thus potentially biasing the results of the study.
7. Some participants did not provide sufficient information for comprehensive causality assessment of reported AEs. Additional information and medical records were requested when possible. However, many reported events remained based primarily on participant self‐report.
8. Despite efforts being made to collect missing data, participants failed to report a substantial proportion of the required information. For many demographic variables, data were missing for 40%–50% of the participants. Missing data is almost universal in longitudinal studies of this nature and, when variables with missing values differ systematically from variables with observed values, bias may be introduced [28]. Missing data may therefore have reduced both the internal validity and interpretability of the study findings.
9. This report is based on a case series focusing solely on exposed pregnancies, and data from a direct comparison group were not available. The COVID‐PR methodology incorporated recruitment of two comparator groups: an active comparator cohort comprising women treated with medications other than RDV specifically indicated for COVID‐19, and an unexposed comparator cohort comprising women hospitalized for COVID‐19 during pregnancy without receipt of any specifically indicated medication. Recruitment for these comparator groups remains ongoing and may support future comparative analyses.

### 4.3. Future Directions

The outcomes from the present study, despite these limitations, contribute to the growing body of knowledge surrounding RDV′s safety profile during pregnancy. As the study continues and additional data are amassed, a more comprehensive understanding of RDV use during pregnancy may become possible.

## 5. Literature Review: Efficacy and Safety of RDV During Pregnancy

### 5.1. Preclinical Data

The multistep metabolic process required to generate the active intracellular nucleotide triphosphate analogue suggests that RDV itself is unlikely to traverse the human placenta at clinically meaningful levels. In contrast, the properties exhibited by the primary metabolites, GS‐441254 and GS‐443902, including their prolonged half‐lives, low molecular weights, and substantial unbound fractions, suggest an increased likelihood of placental transfer [5, 6]. This has been demonstrated in studies of experimental RDV administration in pregnant rats. In maternal blood, RDV remained detectable for only 40 min before being metabolized into GS‐441524, which subsequently sustained elevated levels within the placenta, fetus, and amniotic fluid for up to 4 h. Although GS‐441524 was detected in the placenta, RDV itself did not make its way into the fetal compartment. Concentrations of GS‐441524 were most prominent in maternal blood, followed by amniotic fluid, fetus, and placenta [5]. Current knowledge of RDV or metabolite concentrations in the fallopian tubes, the site of preimplantation development, remains unknown [4].

Animal investigations involving RDV have not revealed genotoxicity or adverse effects on embryo‐fetal or peripostnatal development [1, 29]. In pregnant rabbits and rats, administration of RDV at doses up to four times higher than those employed in human treatments did not result in detrimental effects on intrauterine growth, survival, or fetal morphology [6].

A study exploring the in vitro development of mouse preimplantation embryos following RDV exposure unveiled significant impairment of embryo development at clinically relevant concentrations. Higher concentrations precipitated the collapse of the blastocyst cavity, whereas lower concentrations hindered the formation of the inner cell mass lineage, though overall morphology remained unaffected. The deleterious effects of RDV were most pronounced when exposure occurred 2.5–3.5 days after embryo formation. In contrast, the RDV metabolite, GS‐441524, had no discernible impact on blastocyst cavity formation or maintenance. Although certain changes in the expression of cell lineage markers were noted, these diverged from the pattern observed with RDV. In vitro human embryonic stem cell viability was also diminished following RDV administration, displaying a dose‐dependent relationship. Exposure to GS‐441524 only modestly affected cell viability, and solely at the highest concentration tested. It remains uncertain whether similar outcomes would manifest in human embryos. Thus, the molecular repercussions of RDV and GS‐441524 on preimplantation development may diverge significantly [4].

### 5.2. Efficacy in the General Population

Following encouraging outcomes observed in nonpregnant individuals, where RDV exhibited a reduction in recovery time among hospitalized adults and a decreased risk of hospitalization and mortality when administered within 7 days of symptom onset [3], RDV was made available for compassionate use in pregnancy on March 21, 2020. The United States Food and Drug Administration authorized emergency use of RDV for patients with severe COVID‐19 on April 16, 2020 [2, 5]. As of May 1, 2020, RDV became available for pregnant women with severe COVID‐19 [2].

### 5.3. Efficacy in Pregnant Patients

Available evidence regarding RDV use during pregnancy consists primarily of small observational studies, case series, and case reports, with few studies including comparator groups.

Available evidence regarding the efficacy of RDV in pregnant patients with COVID‐19 is summarized in Table 3. Data were available for a total of 265 patients from individual studies, as well as a systematic review [10] that focused on 113 of these patients. Four cohort studies [2, 3, 9, 30] accounted for 187 patients, with the remaining 78 coming from multiple small case series [22–24, 27, 34, 43, 44] or single case reports [25, 31–33, 35–42].

**Table 3:** Summary of the evidence of the efficacy of remdesivir treatment in pregnant patients.

Author, year, reference	Number of subjects treated with RDV	Main findings
**Systematic reviews**		
Budi, 2022, [10]	113	RDV improved the clinical condition of pregnant patients with COVID‐19, especially those with a better clinical status at baseline and who received RDV earlier
**Cohort studies**		
Burwick, 2021, [2]	67	All subjects were hospitalized with severe COVID‐19
		93% of subjects recovered by Day 28 of follow‐up
		Highest rates of clinical improvement (98%) were observed in pregnant women who did not require IMV
		Median time to recovery in pregnant women was 5 days for those not on IMV and 13 days for those on IMV (*p* < 0.001)
Eid, 2022, [3]	41:24 in early treatment group (< 7 days from symptom onset); 17 in late treatment group (7+ days from symptom onset).	Patients in early treatment group were significantly less likely than those in late treatment group to be admitted to ICU (21% vs. 59%; OR 0.18; *p* < 0.05) or progress to critical disease (12% vs. 41%; OR 0.20; *p* < 0.05, and had shorter hospital stays (5 days vs. 11 days, *p* < 0.01)
		Maternal deaths numbered 0 in the early treatment group and 2 in the late treatment group

Tavakoli, 2023, [30]	54	RDV‐treated group had significantly longer hospital stay than nonRDV‐treated group (*p* < 0.0001). However, this may reflect institutional policies requiring minimum stay for pregnant patients irrespective of clinical improvement
		Other outcomes (severe COVID‐19, admission to ICU, intubation, cardiopulmonary resuscitation, COVID‐19 related death) did not significantly differ between the two groups
		Positive PCR test for COVID‐19 was noted in 10.2% of infants born to nonRDV‐treated mothers, compared to 1.9% of infants born to RDV‐treated mothers (*p* = 0.04), raising the possibility that RDV treatment may influence vertical transmission risk, although confirmation in larger studies is needed

Brooks, 2024, [9]	25	All participants received at least 1 other medication
		Median hospital stay was 10 days in pregnant participants compared to 7 days in nonpregnant participants
		Clinical deterioration occurred in 3 pregnant patients; 1 required mechanical ventilation for 3 days and 2 required vasopressor or inotropic support for 4 days
**Case reports/series**		
Anderson, 2020, [31]	1	Patient recovered relatively quickly following initiation of RDV treatment
Easterlin, 2020, [32]	1	Patient initially improved following delivery but experienced respiratory complications that ultimately required tracheostomy
Igbinosa, 2020, [22]	3	All patients receiving supplemental oxygen had resolution of this requirement after RDV treatment
Maldarelli, 2020, [33]	1	Patient′s supplemental oxygen requirement decreased steadily following initiation of RDV treatment; after hospital discharge patient still complained of cough and dyspnea on exertion but was significantly improved from admission
		Patient subsequently made full recovery
McCoy, 2020, [34]	5	All patients recovered
Naqvi, 2020, [35]	1	Patient recovered
Schnettler, 2020, [36]	1	Patient improved slowly but still required oxygen on HD17
Chinen, 2021, [37]	1	Patient experience respiratory failure the day after RDV treatment but subsequently recovered after mechanical ventilation/ECMO
Dande, 2021, [38]	1	Patient showed steady improvement following initiation of RDV treatment
Jacobson, 2021, [39]	1	Patient did not improve after RDV treatment
Saroyo, 2021, [23]	2	Both patients recovered
Singh, 2021, [24]	2	Both patients recovered
Yamamoto, 2021, [25]	1	Patient recovered
Alavi, 2022, [40]	1	Patient recovered
Dehghan, 2022, [41]	1	Patient recovered
Hayashi, 2022, [42]	1	Patient recovered
Marzban‐Rad, 2022, [43]	23	All patients recovered

Nasrallah, 2022, [44]	24	17 women treated with RDV within 48 h of hospitalization; all achieved clinical recovery by HD7
		7 women treated with RDV > 48 h after hospitalization; all required supplemental oxygen by HD7
		Women treated with RDV achieved a higher rate of clinical recovery by HD7 than those treated with antibiotics (15/15 vs. 3/11; *p* < 0.001)
		Delaying RDV by 48 h after admission had an inverse association with clinical recovery (HR 2.32, 95% CI 1.45–4.16)

Pagan, 2022, [27]	7	All patients were critically ill at RDV initiation
		All patients recovered

Abbreviations: CI, confidence interval; ECMO, extracorporeal membrane oxygenation; HD, hospital day; HR, hazard ratio; ICU, intensive care unit; IMV, invasive mechanical ventilation; OR, odds ratio; PCR, polymerase chain reaction; RDV, remdesivir.

In the cohort studies, the majority of patients recovered after RDV administration. However, in one study [2], 7% of patients had not recovered by Day 28 of follow‐up and in another [9], clinical deterioration occurred in three pregnant patients compared with one patient in the nonpregnant comparator group; among the pregnant patients, one required mechanical ventilation for 3 days, whereas two required vasopressor or inotropic support for 4 days. Hospital stay was increased in patients treated with RDV in another of the cohort studies, but the authors suggested that this may reflect institutional policies requiring a minimum duration of inpatient monitoring for pregnant patients receiving antiviral therapy, irrespective of clinical improvement. Other clinical outcomes, including intensive care unit admission, need for intubation, cardiopulmonary resuscitation, and COVID‐19–related mortality did not differ between treatment groups in this study [30]. Conversely, hospital stays were shorter, and admission to ICU or progression to critical disease were less likely in patients treated with RDV within 6 days of symptom onset, compared to those treated 7–14 days after onset [3].

Only two patients in the case series/reports did not recover after RDV treatment. One initially showed improvement following delivery of her baby but then experienced respiratory complications and ultimately required a tracheostomy [32], whereas the other did not respond to RDV therapy [39]. In addition to these, cessation of symptoms was slow in a third patient, who still required oxygen on the 17th day of hospitalization [36]. However, there was some evidence that women treated with RDV for moderate COVID‐19 symptoms achieved a higher rate of clinical recovery by Day 7 of hospitalization than those receiving glucocorticoids and/or antibiotics. Furthermore, patients who underwent RDV treatment within 48 h of hospital admission displayed superior recovery rates relative to those who received the intervention later [44]. However, these findings should be interpreted cautiously given the limited sample sizes, lack of comparator groups in most studies, and potential coadministration of other therapies.

Additionally, there is limited evidence suggesting that administration of RDV during pregnancy may be associated with a reduced risk of vertical transmission of SARS‐CoV‐2, with infants born to treated mothers demonstrating a lower incidence of positive polymerase chain reaction test results in one study [30].

### 5.4. Safety in Pregnant Patients

Published safety data are largely derived from observational cohorts and case reports involving second‐ and third‐trimester exposures, with limited information available regarding first‐trimester exposure.

Available information regarding the safety of RDV use during pregnancy remains limited [1, 10]. The existing data are summarized in Table 4. As with the efficacy literature, most studies were small, with the largest being based on only 67 patients; much of the evidence comes from case series or single case reports.

**Table 4:** Summary of the evidence of the safety of remdesivir (RDV) treatment in pregnant patients.

Author, year, reference	Number of subjects treated with RDV	Main findings
**Systematic reviews**		
Jorgensen, 2021, [6]	156	Includes women treated with RDV for Ebola virus and COVID‐19 infection
		13 adverse pregnancy outcomes were reported: 7 spontaneous abortions, 2 induced abortions, and 4 stillbirths; 9 of these events occurred in women treated for Ebola virus
		Five cases of congenital abnormalities were also identified. In all cases, RDV was received after the first trimester of pregnancy

Budi, 2022, [10]	113	Most deliveries were by cesarean section, primarily due to emergency causes
		Total incidence of AEs was 29% for those receiving 5‐day course of RDV, rising to 38.5% for patients receiving 10‐day course
		Commonest AE was transaminitis, which was more likely after 10‐day course of RDV than 5‐day course; 2 patients had raised enzyme levels before RDV treatment
		There were no maternal deaths

Romao, 2024, [45]	Not available	Review of all adverse drug events reported to US FDA Adverse Event Reporting System from 2020 to 2022.
		Abortion, fetal death, premature labor, and premature delivery were identified as potential safety signals.
**Cohort studies**		
Pierce‐Williams, 2020, [46]	16	All patients had severe or critical COVID‐19.
		High rates of preterm delivery, low birth weight, cesarean delivery and admission to NICU were reported. However, these endpoints related to the entire group of patients, many of whom did not receive RDV.

Burwick, 2021, [2]	67	One‐third of women reported an AE, and 18% reported a serious AE.
		Most commonly reported AEs were related to pregnancy and underlying disease.
		1 spontaneous abortion occurred in a woman with a history of intravenous drug use, who was subsequently found to have concurrent *Staphylococcus aureus* bacteremia, tricuspid valve endocarditis, and septic arthritis.
		19 of the 26 (73%) of the observed deliveries were by cesarean section; 89% of which were emergent.
		1 maternal death occurred due to underlying COVID‐19 disease.
		Grade 3 elevations of ALT and AST occurred in 9% and 5% of pregnant women, respectively. There were no Grade 4 elevations.
		Seven women discontinued RDV: 5 due to elevations in liver enzymes, 1 due to nausea, and 1 due to hemoptysis.
		The investigators concluded that RDV was generally well tolerated within the limitations of the study.

Eid, 2022, [3]	41:24 in early treatment group (< 7 days from symptom onset); 17 in late treatment group (7+ days from symptom onset).	Occurrence of most adverse pregnancy outcomes (gestational diabetes, hypertensive disorders of pregnancy, cesarean delivery, postpartum hemorrhage, preterm delivery for both obstetric indications and due to worsening COVID‐19) were not affected by the interval between symptom onset and RDV treatment.
		Preterm delivery for worsening COVID‐19 was significantly less frequent in the early treatment group compared to the late treatment group (5% vs. 33%; *p* = 0.05).
		2 maternal deaths occurred in late treatment group, both of which were attributed to COVID‐19, not RDV treatment.

Gutierrez, 2022, [26]	39	16.7% of patients discontinued RDV due to transaminitis.
		No other adverse reactions were recorded.

Tavakoli, 2023, [30]	54	Maternal incidence of gestational diabetes, preeclampsia, and abortion did not significantly differ between the RDV‐treated and nonRDV‐treated groups.
		The incidence of preterm labor did not differ between the RDV‐treated and nonRDV‐treated groups.
		The incidence of both NICU admission and neonatal death was reduced in the RDV‐treated group compared to the nonRDV‐treated group, but neither reached statistical significance.

Brooks, 2024, [9]	25	AEs occurred during treatment period in 62.5% of pregnant patients.
		During 4 weeks of follow‐up, serious AEs occurred in 23.8% of pregnant patients.
		1 participant reported intrauterine fetal demise at 26.7 weeks gestation.
		During delivery, 3 mothers experienced AEs, of which 2 were Grade 3 or higher. There was 1 case each of fetal heart deceleration and maternal respiratory failure.
		Of the 16 live births, 4 (25%) were delivered prematurely and 2 (12.5%) were small for gestational age.
		No AEs or adverse pregnancy outcomes were deemed related to RDV treatment.

**Randomized controlled studies**		
Mulangu, 2019, [47]	6	Study of RDV in patients with Ebola virus from which pregnant patients were not excluded.
		No AEs were reported in the mothers and there was no evidence of fetal toxicity.
**Case reports/series**		
Anderson, 2020, [31]	1	Mild transaminitis was noted following RDV treatment.
		Patient had no further issues after discharge from hospital.

Easterlin, 2020, [32]	1	Mother developed preeclampsia.
		Very low birthweight infant born very preterm by Cesarean delivery.
		Infant remained hospitalized due to Grade 2 intraventricular hemorrhage, patent ductus arteriosus, and had findings suggestive of tuberous sclerosis.

Igbinosa, 2020, [22]	3	1 patient discontinued treatment due to development of transaminitis.
		No other AEs were reported.
Maldarelli, 2020, [33]	1	Mild transaminase was reported which peaked on Day 4 of RDV treatment, before returning to below‐admission levels No other AEs were reported.

McCoy, 2020, [34]	5	1 patient discontinued treatment due to elevated aminotransferases attributed to RDV treatment.
		3 of the 4 observed deliveries were by cesarean section.
		All 4 deliveries occurred before 37 weeks of gestation.

Naqvi, 2020, [35]	1	No AEs reported.
Schnettler, 2020, [36]	1	Patient required Cesarean delivery; no other AEs reported.
Chinen, 2021, [37]	1	Infant was delivered by preterm cesarean section and admitted to NICU.
Dande, 2021, [38]	1	No AEs reported.
Jacobson, 2021, [39]	1	Infant was delivered preterm by cesarean section and admitted to NICU.
		Infant had low birthweight and adrenal insufficiency; latter likely due to dexamethasone administered to mother to treat COVID‐19.
Saroyo, 2021, [23]	2	1 delivery was by cesarean section due to previous cesarean section.
		No other AEs were reported.

Singh, 2021, [24]	2	1 patient experienced elevated ALT and AST levels; these resolved within 2 weeks without the need to discontinue RDV treatment.
		1 preterm birth reported.
		1 Cesarean delivery, due to fetal distress, reported.
		No other AEs were reported.

Yamamoto, 2021, [25]	1	No AEs reported.
Alavi, 2022, [40]	1	Infant was delivered preterm by cesarean section and admitted to NICU; discharged after 10 days in normal condition.
Dehghan, 2022, [41]	1	No AEs reported.
Hayashi, 2022, [42]	1	No AEs reported.
Marzban‐Rad, 2022, [43]	23	Out of 6 deliveries, 5 were by cesarean section. Reasons were drug allergy and sensitivity to RDV, patient choice, fetal descent, and fetal distress. No reason was given for the fifth case.
Nasrallah, 2022, [44]	24	Incidental oligohydramnios occurred in 3 (12.5%) women within 5 days of completing RDV treatment.
		Elevated transaminases occurred in 8 (33.3%) of women treated with RDV.
Pagan, 2022, [27]	7	There was 1 intrauterine fetal demise.
		Two women were delivered due to respiratory distress.
		No AEs were associated with RDV treatmen.t

Abbreviations: AE, adverse event; ALT, alanine aminotransferase; AST, aspartate transaminase; NICU, neonatal intensive care unit; RDV, remdesivir; US FDA, United States Food and Drug Administration.

The earliest insights stem from a randomized controlled study involving patients with Ebola virus, encompassing six pregnant women in the RDV treatment group. This study reported no adverse effects in the mothers or indications of fetal toxicity linked to RDV treatment [47].

A large review [45] of all AEs associated with the use of RDV reported to the US FDA Adverse Event Reporting System between 2020 and 2022 identified abortion, fetal death, premature labor, and premature delivery as potential safety signals. However, the authors did acknowledge that such signals do not, in isolation, establish causality or confirm an increased risk. A smaller review [6], based on data from Gilead′s global safety database, also reported several instances of spontaneous abortion and stillbirth, with many of these events occurring in women treated with RDV for Ebola virus infection, rather than COVID‐19. Five cases of congenital abnormalities were also identified, although in every case, RDV was administered after the first trimester of pregnancy. The final review [10] reported that cesarean delivery was common in women treated with RDV, although this was primarily due to emergency causes. Overall, AEs were common and increased with the duration of RDV treatment, with transaminitis being the most frequently reported event.

AEs occurred in up to 63% of women who participated in cohort studies, and were serious in up to 24%, although many of these were due to pregnancy and underlying disease, rather than RDV treatment [2, 9]. Transaminitis was frequently reported and led to discontinuation of treatment in at least 11 cases [2, 26]. Similarly, there was a high rate of cesarean deliveries [2, 46], although in one study this finding applied to the entire study group, many of whom did not receive RDV [46]. This study also reported increased rates of preterm delivery, low birthweight and admission to the neonatal intensive care unit. High rates of preterm birth and small for gestational age were reported among RDV‐treated patients in one study [9], although elsewhere, RDV treatment had no significant effect on the incidence of any maternal and neonatal outcome [30]. In addition, the timing of RDV treatment did not appear to affect the incidence of most adverse pregnancy outcomes, with the exception of preterm delivery due to worsening COVID‐19, which was significantly reduced in women treated within 7 days of symptom onset [3]. Three cases of maternal death were reported overall, with underlying COVID‐19 infection being cited as the cause for all of them [2, 3]. Other serious events, including spontaneous abortion [2], intrauterine fetal demise, fetal heart deceleration, and maternal respiratory failure [9], were reported as isolated cases.

AEs identified in the case series/reports followed a similar pattern to those reported in the cohort studies. Transaminitis was relatively frequent [22, 24, 31, 33, 34, 44] and two women discontinued RDV because of it [22, 34]. Multiple cases of preterm delivery [24, 27, 32, 34, 37, 39, 40] and cesarean delivery [23, 24, 32, 34, 36, 37, 39, 40, 43] were also reported, although low birthweight was less common [32, 39]. One mother developed preeclampsia [32] and incidental oligohydramnios was reported in three women [44]. Four babies were admitted to the NICU [32, 37, 39, 40]. One remained hospitalized due to Grade 2 intraventricular hemorrhage, patent ductus arteriosus, and findings suggestive of tuberous sclerosis [32], whereas another had adrenal insufficiency, likely due to dexamethasone administered to the mother to treat her COVID‐19 infection [39]. A single case of intrauterine fetal demise was reported [27].

### 5.5. The Impact of Maternal Comorbidities

It is important to acknowledge that a significant proportion of these pregnant women were severely ill due to COVID‐19, a condition in itself associated with an elevated risk of adverse pregnancy outcomes [48]. Some patients may have received treatments other than RDV, such as steroids, anticoagulants, hydroxychloroquine, antibiotics, and convalescent serum [27, 37, 43, 46]. Furthermore, numerous women requiring hospitalization for COVID‐19 may already possess a high‐risk pregnancy due to underlying medical conditions [2]. Consequently, the precise attribution of these AEs to RDV treatment, COVID‐19 infection, shared risk factors, or a confluence of these elements remains uncertain. A comparison of pregnancy outcomes between women treated with RDV and those receiving other medications showed comparable rates of preterm delivery in both groups [48]. In light of the well‐established association between respiratory compromise, hypoxia, and preterm delivery, this finding suggests that the increase in preterm delivery might be primarily influenced by COVID‐19 infection rather than RDV treatment [2, 48]. A notable number of emergency cesarean sections executed for maternal or fetal obstetric reasons lead to preterm delivery [2, 10]. Accordingly, it is conceivable that a substantial portion of cesarean deliveries may stem from COVID‐19 infection accompanied by respiratory compromise, rather than RDV treatment. As of now, only one documented cesarean delivery has been explicitly attributed to “drug allergy and sensitivity to remdesivir” [43].

Overall, the available literature on RDV use during pregnancy remains limited and is derived predominantly from observational studies, compassionate use programs, and case reports. Although no consistent pattern of drug‐related maternal, fetal, or neonatal toxicity has emerged, interpretation is constrained by small sample sizes, incomplete follow‐up, heterogeneous patient populations, variable disease severity, and frequent absence of comparator groups.

## 6. Overall Conclusions

In conclusion, our analysis of outcome and safety data derived from eight exposed pregnancies with follow‐up data did not identify a consistent pattern suggestive of a specific safety concern. Given the considerable risks associated with untreated COVID‐19, particularly during pregnancy, available evidence remains insufficient to draw definitive conclusions regarding safety. Further evidence from larger, well‐designed comparative studies is required to better characterize the safety and effectiveness of RDV use during pregnancy.

## Author Contributions

All listed authors have made substantial contribution to this study. Diego F Wyszynski: Conceptualization, Methodology, Writing – Original Draft, Writing – Review and Editing, Supervision, Funding and acquisition. Cheryl Renz: Methodology, Validation, Investigation, Writing – Original Draft, Writing – Review and Editing, Supervision, and Project administration. Aprille Espinueva: Methodology, and Writing – Review and Editing. Lee P Shulman: Investigation, Resources, Writing – Original Draft, and Writing – Review and Editing. Eun Young Lee: Methodology, and Writing – Review and Editing. Andrew Fitzgibbon: Validation, Investigation, Writing – Review and Editing, Supervision, and Project administration. Gina Brown: Methodology, Writing – Original Draft, and Writing – Review and Editing.

## Funding

This study was supported by the Gilead Sciences, 10.13039/100005564; GlaxoSmithKline, 10.13039/100004330; Regeneron Pharmaceuticals, 10.13039/100009857; Merck Sharp and Dohme, 10.13039/100009947; Roche, 10.13039/100004337; and Genentech, 10.13039/100004328.

## Disclosure

All authors have read and approved the final version of the manuscript. Diego Wyszynski had full access to all of the data in this study and takes complete responsibility for the integrity of the data and the accuracy of the data analysis.

## Ethics Statement

Diego Wyszynski affirms that this manuscript is an honest, accurate, and transparent account of the study being reported; that no important aspects of the study have been omitted; and that any discrepancies from the study as planned (and, if relevant, registered) have been explained.

## Conflicts of Interest

D.F.W., C.R., and A.F. are employees of Thermo Fisher Scientific, a company that receives funding from Gilead Sciences and various other pharmaceutical companies. L.P.S. reports no conflicts of interest. A.E., E.L., and G.B. are employees of Gilead Sciences.

## Data Availability

The data that support the findings of this study are available on request from the corresponding author. The data are not publicly available due to privacy or ethical restrictions.
